# Very High Incidence of *Chlamydia trachomatis*, *Neisseria gonorrhoeae*, and *Treponema pallidum* among Low-Risk MSM in an Outpatient Clinic in Wroclaw, Poland in 2019–2020

**DOI:** 10.3390/ijerph20032582

**Published:** 2023-01-31

**Authors:** Bartosz Szetela, Łukasz Łapiński, Katarzyna Giniewicz

**Affiliations:** 1 Department of Infectious Diseases, Liver Disease and Acquired Immune Deficiencies, Wroclaw Medical University, 50-367 Wroclaw, Poland; 2 Faculty of Veterinary Medicine, Wroclaw University of Environmental and Life Sciences, 50-375 Wroclaw, Poland; 3 Statistical Analysis Centre, Wroclaw Medical University, 50-367 Wroclaw, Poland

**Keywords:** STI, PrEP, HIV, syphilis, *Chlamydia trachomatis*, *Neisseria gonorrhoeae*

## Abstract

Background: The rise in sexually transmitted infections and chemsex has led to syndemy with HIV, partly due to common routes of transmission and clustered transmissions. Despite this, barriers to STI care and PrEP still remain. We sought to determine whether MSM at low risk for HIV infection were also at low risk for other STIs. Methods: The study group was tested for HIV, HCV, and *Treponema pallidum*, as well as had urethral, rectal, and oropharyngeal smears performed for *Neisseria gonorrhoeae* (NG) and *Chlamydia trachomatis* (CT) six months apart. The control group was tested once to define the background incidence. Results: *Treponema pallidum*, CT, and NG prevalence was very high at both time points and was similar to the control group. CT was especially common in the control group (20.58%) and the study group at the rectal site at the second time point (9.37%). NG dominated the oropharyngeal site (15.87%), with urethral site sparing. NG infection was associated with an increased number of partners, not condom use (OR, 1.082 [95% CI; 1.009–1.171]). Risk behavior did not change between the time points. *Treponema pallidum*, CT, and NG incidence was exceptionally high (12.5/100PY, 25.39/100PY, 34.92/100PY, respectively; pooled 87.5/100PY) and was comparable to other studies of high-risk MSM. Conclusions: Despite a lower risk for HIV acquisition, the study group was at a very high risk for other STIs, and this risk remained high throughout the study. Patients and medical professionals should be aware of syphilis, gonorrhea, and chlamydiosis transmission risks, and screening should be performed accordingly. Prophylactic programs need to be updated to specifically include lower-risk individuals.

## 1. Introduction

STIs (sexually transmitted infections) have been on the rise worldwide, and because of their common routes of transmission, they have become intertwined with HIV, leading to syndemy among MSM (men who have sex with men), irrespective of PrEP use (pre-exposure prophylaxis) [1,2,3]. Condom use decline and oral sex are thought to be at the core of this rise [4], while most *Chlamydia trachomatis* (CT), *Neisseria gonorrhoeae* (NG), and syphilis infections being asymptomatic or self-limiting among MSM makes timely diagnosis even more difficult [5,6].

Guidelines state that all individuals at substantial risk of HIV infection and interested in PrEP should receive it. However, substantial risk has not been clearly defined. Medical professionals should be aware of subtle or occasional risk scenarios and be able to discuss them with their patients [7,8,9], despite fear of increased STI incidence during PrEP use [10,11]. Data on the prevalence and incidence of STIs among PrEP users are mixed. Early trials showed no risk compensation, whereas later trials showed a slight increase in STI incidence, mainly due to the increased number of partners (not condom use) and among a subgroup of younger patients [2,4,11,12,13,14,15,16].

Stratification to low- and high-risk behavior has been at the core of prophylactic interventions, in part due to the cost of public programs [8]. High-risk individuals receive full access to available prophylactic measures, including HIV and other STIs testing, condom provision, drug addiction treatment, and PrEP. However, low-risk individuals might not be getting access to the required prophylaxis due to their personal risk scenarios changing over time, not being aware of their actual risk, or simply not being ready to reveal it during their first few visits [17,18].

We wanted to analyze the prevalence of selected STIs among lower-risk MSM compared to seemingly higher-risk MSM from a local voluntary counselling and testing site (VCT), examine incident infections six months later, and measure risk behavior change in this period. To our knowledge, this is the only prospective and longitudinal study among MSM in Poland so far, and one of the few dealing with the subject in the world.

## 2. Materials and Methods

The study took place between December 2019 and December 2020 at the municipal outpatient All Saint’s Clinic in Wroclaw (Poradnia Wszystkich Swietych), Lower Silesia region, Poland, and included 64 low-risk MSM who were excluded from two prophylactic trials (i.e., STIPnet and MOSAICO) due to too low risk. Low risk was defined as having fewer than two risky sexual contacts in the last 6 months—that is, anal intercourse without condoms with partners not trusted or tested, not on PrEP, and not with an undetectable viral load if HIV infected.

The control group of 103 random MSM from the local VCT received one-time testing for all STIs. VCT patients are considered to be at a high risk for HIV infection. During the trial, HIV prevalence among all VCT MSM was 2.46%, that of syphilis was 4.77%, and that of HCV was 0.61% (annual Wroclaw VCT report, not published).

Patients were recruited at the All Saint’s Clinic during regular medical visits from among PrEP users or STI patients referred from local VCT sites. During the first visit, HIV, HCV, syphilis, CT, and NG tests were performed and treatment was given if necessary. Treatment included benzatine penicillin 2.4MU i.m. once for syphilis, ceftriaxone 1 g i.m. plus 2 g azithromycin p.o. once for gonorrhea, and azithromycin 1 g p.o. once or doxycycline 100 mg p.o. twice a day for 7 days in the case of chlamydiosis. Patients filled out anonymous risk behavior questionnaires online (tablet available). The same tests and questionnaire were repeated six months later.

HIV/p24 testing was performed using the Roche Elecsys HIV Duo tests on the COBAS machine. Confirmation testing was performed with the Geenius HIV 1/2 Confirmatory Assay (BIO-RAD).

HCV screening was performed using CE-registered rapid tests by INFO Turklab (REF 1434) for whole blood. To confirm positive rapid test results, real-time PCR on an AlinityM machine by Abbott was performed using a 4J8690 m2000 RealTime RNA HCV Amplification Reagent Kit (PCR test) and 4J8670 m2000 RealTime HCV Calibrator Kit with 4J8680 m2000 RealTime HCV Control Kit.

Syphilis screening was performed using rapid tests by Beright (REF ISY-402) for whole blood. Specific tests were used for confirmation: TPHA (Plasmatec—passive hemagglutination), FTA and FTA-Abs (indirect immunofluorescence tests from Venereal Research Institute in Bialystok Poland), and VDRL titers (BIOMED). Seroconversion with specific treponemal antibodies present or rising VDRL titers in patients with a previous syphilis diagnosis confirmed early syphilis.

CT and NG swabs were performed with dedicated Abbot multi-Collect specimen collection kits and tested at Diagnostyka Medical Laboratories with Real-Time PCR on m2000rt by Abbott using 2G2891—m2000 RealTime *Chlamydia trachomatis*/*Neisseria gonorrhoeae* Amplificaction Reagent Kit (PCR test) and 2G2880 m2000 *Chlamydia trachomatis*/*Neisseria gonorrhoeae* Control Kit. In one patient, the results were returned as invalid, and hence not included in the analysis.

All of the above tests were CE-registered for diagnostic use in humans.

The questionnaire included questions on age, education, sexual contact, condom use depending on the type of sex, number of partners in the last 6 months, presence of a stable partner, chemsex, PreP, PEP, and antibiotic use. To minimize the risk of forced false answers, the patients had the possibility to omit some questions, as a result decreasing the number of available responses to compare between timepoints.

Statistical analyses were performed using the R software version 4.0.4. *p*-values for the difference in distribution between the first and second time points were obtained using McNemar’s test. For differences between groups (not paired), Fisher’s exact test was used. The difference in relationship length between time points (paired) was verified using the Wilcoxon test after confirming normality with the Shapiro–Wilk’s test and symmetry with the Miao–Gel–Gastwirth test. A permutation-based test of medians was used to compare medians when the assumption of symmetry was not met. Anderson–Darling’s test for the four groups was used to examine the differences in the number of partners between age groups. Simple logistic regression models were built to obtain odds ratios (OR) for dependence on prevalence based on the number of partners in the last six months. All the tests were conducted at a significance level of 0.05.

This trial received Wroclaw Medical University Ethics Committee review number KB-488/2019.

## 3. Results

### 3.1. Social and Behavioral Data

The age distribution of the studied group was similar to that of the controls in almost all strata, with only the youngest age group, 18–24 years old, being overrepresented in the control group (10.93 vs. 28.15%; *p* = 0.0012).

For a comparison of the demographics between the comparator and study groups, see Table 1.

During the observation period, the number of stable relationships increased in the study group, while previous relationships lasted an additional six months (Wilcoxon test *p* = 0.6425), showing a stable relationship status and looking for new relationships if single. The number of patients reporting unknown sexual partners did not change during the trial, as did the median number of sexual partners. There was no statistical difference between the time points (*p* = 0.5192, Wilcoxon test with symmetry assumption) or study group vs. control (1. timepoint: *p* = 0.437, 2. timepoints: *p* = 0.4119, permutation-based test of medians), even with age stratification (1. timepoint: *p* = 0.3924, 2. timepoint: *p* = 0.09165, Anderson–Darling test for four groups).

Overall, 22 patients (59.5% of the patients who had given answers at both timepoints) had more than two anal contacts without condoms with two or more partners in the last six months. This number did not change during the trial (*p* = 0.72), nor the type of anal and oral contacts (receptive/insertive/universal). The same stable risk scenarios were observed for sex under the influence of alcohol or drugs (42% vs. 39%; see Table 2—the percentage values are the result of the actual number of responses in the questionnaire at both timepoints without responses missing).

PrEP use increased substantially from 66.6% to 85.7% at the second time-point (*p* = 0.043).

### 3.2. STI Prevalence and Incidence

In the control group, two HIV infections (1.94%), eight syphilis infections (7.76%), and one HCV infection (0.97%) were confirmed. Oropharyngeal NG was the most common, followed by rectal and urethral NG (10.78%, 7.84%, and 5.88%, respectively). Oropharyngeal, urethral, and rectal CT infections were spread evenly (9.9%, 8.82%, and 10.89%, respectively), with a high pooled prevalence of 20.58%.

In the study group, there were no confirmed HIV infections, either during the first or during the second visit. Hepatitis C was diagnosed in one patient at the first time point (1.56%), and no infections were confirmed later. Syphilis was confirmed in two patients (3.12%) at screening and four at the second time point (6.25%, incidence 12.5/100PY). The prevalence increased from 34.37% at study entry to 43.7%, with an incidence as high as 87.5/100PY.

Oropharyngeal NG was the most common, with increasing prevalence over the course of the study (9.52% vs. 15.87%, *p* = 0.38); however, this increase was statistically insignificant. The highest NG incidence of 31.74/100PY was seen at the oropharyngeal site, followed by rectal and urethral sites. The pooled NG incidence was 34.92/100PY.

CT diagnoses were much less common and were concentrated at the rectal site, especially at the second time point (9.37%; incidence, 18.75/100PY). Other sites of infection, especially at study entry, were rare, as opposed to the control group, where CT was very common at all sites. The pooled CT incidence was 25.39/100PY.

**Figure 1:**
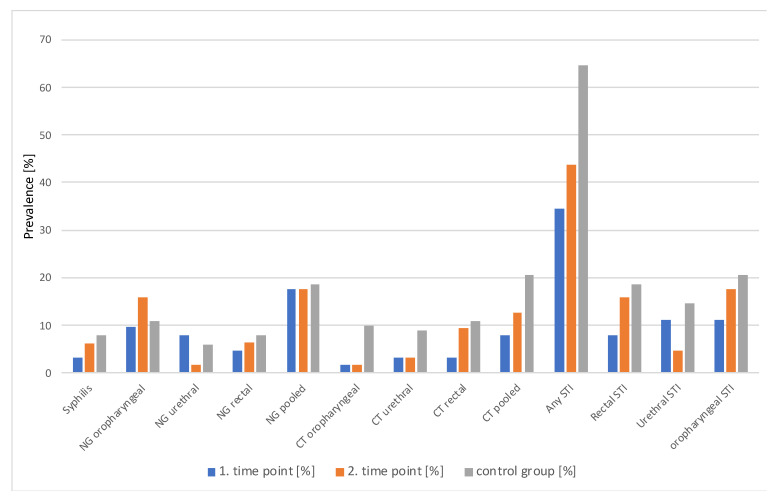
STI prevalence by group, timepoint and anatomical site.

Overall, 79% of CT and NG infections in the study group and 84% in the control were asymptomatic. For STI prevalence and incidence data, see Figure 1 below and Table 3.

Although CT infections seemed to be more common in the control group and oropharyngeal NG at the second time point, no statistically significant differences were observed, irrespective of whether the sites of infection were analyzed separately or pooled between time points (McNemara test) or between groups (Fisher test). Even when all HIV, HCV, syphilis, NG, and CT infections were pooled together, there was no difference in prevalence between groups or time points (see Table 3). 

### 3.3. Correlation of Infections with Socio-Behavioral Data

The Fisher test was used for categorical variables, while the Wilcoxon test was used for continuous variables.

At study entry, syphilis infection was correlated with younger age (*p* = 0.029), oropharyngeal gonorrhea was correlated with higher education (*p* = 0.023) and a higher number of partners (*p* = 0.009), while rectal chlamydiosis was correlated with all sexual partners being known (*p* = 0.008). At the second time point, only oropharyngeal NG was correlated with active oral contact (*p* = 0.04). These differences were not statistically significant after the Bonferroni correction. 

**Figure 2:**
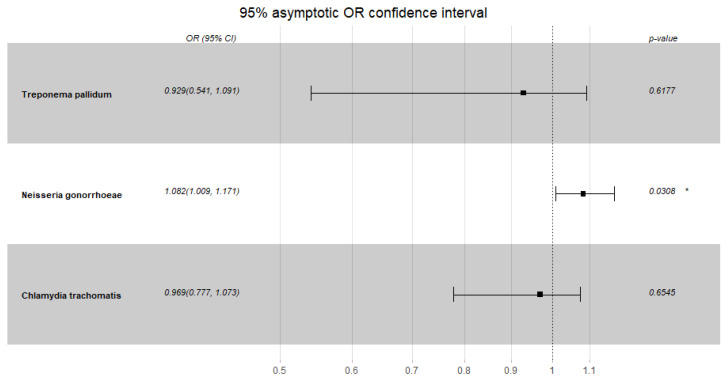
*Neisseria gonorrhoeae*, *Chlamydia trachomatis* and *Treponema pallidum* risk of infection in the studied group dependent on the number of sexual partners—first timepoint. * *p* < 0.05.

Condom use in the study group was rare, probably due to the low number of unknown sexual partners; hence, there was no correlation with any STI infection, even in the oropharyngeal region (*p* = 1). However, the probability of NG infection at study entry increased with an increase in the number of partners (OR, 1.082 [95% CI; 1.009–1.171])–see Figure 2 below. No such correlation was observed for the other pathogens, time points, or controls.

## 4. Discussion

Despite lower risk for HIV acquisition, the group was at high risk for *Treponema pallidum*, NG, and CT infections. The risk did not change during the trial period. Access to testing, treatment, and PrEP did not lead to risk compensation. However, we observed a subgroup of younger patients with an exceptionally high number of partners and NG infection or syphilis. Common oral contact without condoms and saliva being used as a lubricant may be responsible for the easy transmission of these STIs. Chlamydia incidence did not increase with increasing number of partners, probably due to the common use of doxycycline and quinolones in this population. This is further supported by the exceptionally high CT prevalence in the control group, who might have had more barriers to medical care. Similar observations were made in the Amsterdam PrEP cohort reported by Hoornenborg et al. [16]. The STI incidence in our study was only slightly lower than that among high-risk PrEP users in the Australian PrEPX study by Traeger et al. [12]. They reported a pooled STI prevalence of 91.9/100PY and a significant increase in the incidence of any STI, especially among younger patients with more partners. The same factors—that is, younger age and number of partners, not condom use—correlated with STI diagnosis in another trial among PrEP users by Golub et al. [19]. These observations are reflected in the CDC’s 2021 guidelines for PrEP provision among MSM, as part of the MSM risk index. They stress that HIV infection risk is the highest among younger patients (18–40 years old), those who had more than six partners during the last six months, those having unprotected anal sex without a condom, and those with HIV-positive partners (although they do not mention U=U messages in this regard) [8]. In our group, only syphilis prevalence was higher among younger patients and NG with an increasing number of partners, which is similar to Hoornenborg et al.’s report [16]. They added older age, previous chemsex use, continuous PrEP at baseline, and previous PEP use as risk factors for receptive condomless anal sex. In our study, lower age was correlated with syphilis, active oral sex, and higher education, and increased number of partners was correlated with pharyngeal gonorrhea while knowing all sexual partners with rectal chlamydiosis. This might be due to lack of knowledge about the pathogens and symptoms of infection, or lack thereof, barriers to medical care as well as possibly cumulative CT infection, and lack of testing in the case of known partner groups. Azarnoosh et al. [20] showed a twofold increase in incident STIs after PrEP initiation among MSM in Denmark, but the baseline STI incidence was close to the incidence seen in our group, including sites of infection.

Jansen et al. reported a slightly lower STI prevalence among MSM in Germany [21]. The overall STI prevalence was 25% among MSM who did not use PrEP (CT, 7.2%; NG, 7.4%) and 40.3% among PrEP users (CT, 13.8%; NG, 14.8%). The infection site distribution showed that the rectal site was the most common for both CT (7.7%) and NG (5.8%). In our group, the most common sites of infection for CT were the rectal/pharyngeal sites, and the pharyngeal site for NG. We ascribe the especially high NG prevalence to the lack of easy access to diagnosis and common NG resistance to quinolones, macrolides, and tetracyclines, which are most commonly used empirically in Poland. Montaño’s report from the MSM PrEP cohort in Seattle similarly showed a reduced number of unknown partners during observation, no change in the number of partners, and a very high prevalence of STIs (49.2%). Montaño noted rising STI prevalence between day 0 and year 1 (35% vs. 49.2%), which mirrors the findings of our group. She suggested that increased testing was responsible for this increase [15]. In our group, however, two-time testing was performed with highly efficacious antibiotic treatment in between when indicated. 

Data from the 2019 meta-analysis by Ong et al. [2] showed a high pooled STI prevalence among PrEP users on day 0 (23.9%), but lower than that in our sample (35.93%). However, the pooled STI incidence of 72.2/100PY was similar to the incidence of 87.5/100PY reported in our study. In the meta-analysis, anorectal infections were most common (CT, 8.5%; NG, 9.3%), with less common urethral (CT, 4.0%; NG, 2.1%) or oropharyngeal sites (CT, 2.4%; NG, 4.9%). We observed universal involvement of all infection sites at recruitment, which may have been the result of a lack of universal testing and treatment approaches for CT and NG in Poland. Problems with a lack of regular STI testing are not country-specific [11].

Surprisingly, even among high-risk MSM from the Discover study, any STI incidence was 99.5/100PY as compared to 87.5/100PY in our study group [22]. The ANRS Prevenir study showed any STI incidence of 66.5–75.5/100PY, chlamydia incidence of 25.3–31.1/100PY, gonorrhea incidence of 33.7–37.8/100PY, and syphilis incidence of 8.5–10.6/100PY [23] similarly to our results (87.5, 25.39, 34.92, and 12.5/100PY, respectively). These data suggest easy and common STI transmission, irrespective of HIV risk.

Overall, 79% of CT and NG infections in our study group were asymptomatic, as in Kenyon’s [24] and Fairley’s [5] reports (90% and 99%, respectively). This stresses the need for screening even if patients report only low-risk behaviors. Kissing, oral sex, or anal sex with condoms when saliva is used as a lubricant [25] are risk factors for syphilis, chlamydiosis, or gonorrhea. In our study, we asked about condom use during anal and oral sex and found no protection from syphilis, NG, and CT infections. Traeger et al. [12] also showed that even consistent condom use was not protective.

An interesting contrasting view to the STI test and treat approach among PrEP users was suggested by Kenyon [24]. He argued that the high STI prevalence among MSM, the dense transmission networks, and low condom use make incidence reduction impossible. The test and treat approach would significantly increase exposure to antibiotics, which would lead to the emergence of resistance while having no clinical benefit [5,6].

The rate of prophylactic doxycycline use was 13–14% in our study, which was higher than the 9% reported by O’Halloran et al. [26]. This high percentage may be due to any antibiotic use being reported by the patients. Nonetheless, prophylactic doxycycline use has been on the rise in Poland in recent years.

## 5. Conclusions

Low-risk MSM may be low-risk only for HIV and HCV infections. Due to the ease of transmission during oral/oro-anal contacts and kissing, the prevalence and incidence of *Treponema pallidum*, NG, and CT are high in this group and follow global trends. Even patients with stable circuits of partners may be at an increased risk of NG, CT, and syphilis and may introduce them to their sexual groups.

Prophylactic programs need to be updated to include the specific needs of lower-risk individuals; however, the testing and treatment frequencies among asymptomatic individuals remain unknown. 

### Limitations

The results from our trial may not be fully representative because the youngest and oldest age groups were underrepresented in the controls. There may also have been a selection bias as patients visiting our clinic may have been more aware of their risk and were hence more open. Patients may also have been mitigating the disclosure of their actual risk during recruitment and filling out the questionnaires to obtain access to free NG and CT testing. The observation period may have been too short to notice changes in behavior, and limited access to medical professionals during the COVID-19 epidemic may have lowered antibiotic exposure. Patients from the control group were included randomly from the pool of VCT patients without individual risk assessment.

## Figures and Tables

**Table 1:** Demographic comparison between groups.

	Control Group	Study Group	*p*
Age 18–24 y.o.; *n* [%*]	29 [28.15]	7 [10.93]	0.0012
Age 25–34 y.o.; *n* [%*]	36 [34.95]	30 [46.87]	1
Age 35–44 y.o.; *n* [%*]	32 [31.06]	20 [31.25]	0.5494
Age 44–54 y.o.; *n* [%*]	6 [5.82]	7 [10.93]	0.3875
Sexual orientation; *n*, [%*] - homosexual - bisexual - heterosexual	99 [96.11] 4 [3.88] 0 [0]	62 [96.87] 1 [1.56] 1 [1.56]	

* Percentage accuracy set at two decimal points, may not sum up to 100%.

**Table 2:** Risk change during observation.

	1st Timepoint	2nd Timepoint	*p*
Currently having stable partner—*n* [%*]	16 [39]	22 [53]	0.041
Number of sexual partners in the last 6 months—*n*	4,5	4	1
Not knowing all sexual partners in the last 6 months—*n* [%*]	27 [75]	26 [73]	0.51
Had more than two anal sexual contacts without condom with at least two partners in the last 6 months—*n* [%*]	22 [59]	22 [59]	0.72
Had more than two oral sexual contacts without condom with at least two partners in the last 6 months—*n* [%*]	33 [91]	29 [80]	0.22
Used PrEP in the last 6 months—*n* [%*]	28 [66]	36 [85]	0.043
Used alcohol/chemsex—*n* [%*]	14 [42]	13 [39]	1
Used antibiotics in the last 6 months—*n* [%*]	8 [15]	6 [14]	>0.05

* Percentage value is the result of actual number of responses in the questionnaire at both timepoints without responses missing.

**Table 3:** Confirmed STIs and comparison between timepoints, groups and anatomical sites.

	Study Group [n]	Infections—First tp [n]	Prevalence First tp [%]	Infections Second tp [n]	Prevalence Second tp [%]	Incidence [/100PY]	Control Group [n]	Infections Control Group [n]	Prevalence Control Group [%]	P (1. tp vs. 2. tp; 1. vs. Control; 2. vs. Control)
HIV	64	0	0	0	0	0	103	2	1.94	NA; 0.1; 0.52
Syphilis	64	2	3.12	4	6.25	12.5	103	8	7.76	0.61; 0.31; 1
HCV	64	1	1.56	0	0	0	103	1	0.97	NA; 0.07; 1
NG										
oropharyngeal	63	6	9.52	10	15.87	31.74	102	11	10.78	0.38; 0.79; 0.34
urethral	63	5	7.93	1	1.58	3.17	102	6	5.88	0.22; 0.75; 0.25
rectal	63	3	4.76	4	6.34	12.69	102	8	7.84	1; 0.53; 1
pooled (positive patients)	63	11	17.46	11	17.46	34.92	102	19	18.62	1; ND; 1
CT										
oropharyngeal	63	1	1.58	1	1.58	3.17	102	10	9.9	0.47; 0.051; 0.052
urethral	63	2	3.17	2	3.17	6.34	102	9	8.82	0.61; 0.2; 0.2
rectal	64	2	3.12	6	9.37	18.75	102	11	10.89	0.44; 0.25; 1
pooled (positive patients)	63	5	7.93	8	12.69	25.39	102	21	20.58	0.5; ND; 0.21
NG + CT										
rectal	63	5	7.93	10	15.87	31.74	102	19	18.62	0.5465; 0.1541; 0.6545
urethral	63	7	11.11	3	4.76	9.52	102	15	14.7	0.7237; 0.4435; 0.1099
oropharyngeal	63	7	11.11	11	17.46	34.92	102	21	20.58	0.6465; 0.1375; 0.6785
Any STI	64	22	34.37	28	43.75	87.5	102	66	64.7	1; 0.0645; 0.09037

tp—timepoint; NG—*Neisseria gonorrhoeae*; CT—*Chlamydia trachomatis*; STI—sexually transmitted infection.

## Data Availability

All data are available from the authors at request.
